# Adrenomedullin protects Leydig cells against lipopolysaccharide-induced oxidative stress and inflammatory reaction via MAPK/NF-κB signalling pathways

**DOI:** 10.1038/s41598-017-16008-x

**Published:** 2017-11-28

**Authors:** Wei Hu, Lei Shi, Ming-yong Li, Pang-hu Zhou, Bo Qiu, Ke Yin, Hui-hui Zhang, Yong Gao, Ran Kang, Song-lin Qin, Jin-zhuo Ning, Wei Wang, Li-jun Zhang

**Affiliations:** 1 Department of Andrology, The First Affiliated of University of South China, No. 69 Chuan Shan Road, Hengyang, 421001 Hunan Province China; 2 Departments of Oncology, Renmin Hospital of Wuhan University, No. 238 Liberation Road, Wuhan, 430060 Hubei Province China; 3 Department of Urology, The First Affiliated Hospital of University of South China, No. 69 Chuan Shan Road, Hengyang, 421001 Hunan Province, China; 4 Department of Orthopedics, Renmin Hospital of Wuhan University, No. 238 Liberation Road, Wuhan, 430060 Hubei Province China; 5 Department of Orthopedics, The First Affiliated Hospital of University of South China, No. 69 Chuan Shan Road, Hengyang, 421001 Hunan Province China; 6 Reproductive Medicine Centre, The First Affiliated Hospital of Sun Yat-sen University, No. 58 Second Zhongshan Road, Guangzhou, 510080 Guangdong Province China; 7 Department of Urology, Renmin Hospital of Wuhan University, No. 238 Liberation Road, Wuhan, 430060 Hubei Province China; 8 Department of Urology, The First Affiliated Hospital of Anhui Medical University, No. 218 Jixi Road, Hefei, 230022 Anhui Province China; 9 Department of Urology, Minda Hospital Affiliated to Hubei Institute for Nationalities, No. 2 Wufengshan Road, Enshi, 445000 Hubei Province China

## Abstract

This study aimed to explore the possible benefits of adrenomedullin (ADM) in preventing oxidative stress and inflammation by using an *in vitro* primary culture model of rat Leydig cells exposed to lipopolysaccharide (LPS). Cell proliferation was detected through CCK-8 and BrdU incorporation assays. ROS were determined with a DCFDA kit, and cytokine concentrations were measured with ELISA assay kits. Protein production was examined by immunohistochemical staining and Western blot, and gene expression was observed through RT-qPCR. Results revealed that ADM significantly reduced LPS-induced cytotoxicity, and pretreatment with ADM significantly suppressed ROS overproduction and decreased 4-HNE and 8-OHdG expression levels and concentrations. ADM pretreatment also significantly attenuated the overactivation of enzymatic antioxidants, namely, superoxide dismutase, catalase, thioredoxin reductase, glutathione peroxidase, glutathione reductase and glutathione-S-transferase. ADM supplementation reversed the significantly increased gene expression levels and concentrations of TNF-α, IL-1β, TGF-β1, MCP-1 and MIF. ADM pretreatment significantly inhibited the gene expression and protein production of TLR-2 and 4. Furthermore, ADM pretreatment markedly reduced the phosphorylation of JNK, ERK 1/2 and p38, phosphorylation and degradation of IκBα and nuclear translocation of p65. Our findings demonstrated that ADM protects Leydig cells from LPS-induced oxidative stress and inflammation, which might be associated with MAPK/NF-κB signalling pathways.

## Introduction

Testes are a part of the reproductive and endocrine systems, and these organs serve as the source of sperm and male sex hormones, which are necessary to maintain normal reproductive function in adult males^1^. Leydig cells, located within the interstitial compartment of the testes, mainly contributed to androgen synthesis and secretion and play an important role in testicular development, normal masculinisation, spermatogenesis maintenance and general male fertility^2^. Infections and inflammation of the male reproductive tract are well-known etiological factors of male subfertility or infertility^3^. In an infected reproductive tract, the innate immune system recruits phagocytic cells and effector molecules to the site of infection by releasing a battery of cytokines and other inflammatory mediators that remarkably affect subsequent events^4^.

Bacterial lipopolysaccharide (LPS), as an active component of Gram-negative bacterial cell walls, contributes to the pathogenesis of bacterial infection in male reproductive tissues^5^. Infection and inflammation can be induced *in vitro* and *in vivo* by administering LPS, and LPS administration in animals inhibits testicular steroidogenesis^6–9^. LPS-mediated production of proinflammatory cytokines exhibits an inhibitory role in Leydig cell function through the production of increased reactive oxygen species (ROS) and consequently disrupt mitochondrial membrane permeability^10–12^. Our previous study demonstrated that LPS-induced inflammation causes oxidative stress and apoptosis in Leydig cells, which may be the major influential factor involved in steroidogenesis impairment^13^. However, the exact underlying mechanisms of oxidative stress and inflammatory reaction by which LPS impairs steroidogenesis are poorly investigated.

Adrenomedullin (ADM) is a 52-amino-acid peptide originally discovered in the tissue extract of human pheochromocytoma and characterised by a potent vasodilatory activity^14^. In addition to a major role in regulating vascular tonus, potent angiogenic, anti-oxidant, anti-inflammatory and anti-apoptotic properties are shown by ADM as an endogenous peptide^15,16^. ADM elicits protective effect against myocardial injury induced by abdominal aortic ischaemia-reperfusion in rats by attenuating oxidative stress and inflammation^17^. Treatment with ADM significantly reduces the development of acute lung injury by downregulating a broad spectrum of inflammatory factors^18^. ADM ameliorates hyperoxia-induced acute lung injury in rats by suppressing oxidative stress and inflammation^19^. ADM deficiency potentiates hyperoxic injury in primary foetal human pulmonary microvascular endothelial cells by increasing oxidative stress and inflammation^20^. ADM2, as a member of the ADM peptide family, causes a restorative effect on steroidogenesis in hydrogen peroxide-treated rat primary Leydig cells^6^. ADM2 may also be considered a promising novel therapeutic target that mitigates diabetic ischaemic heart injury by reducing oxidative stress, inflammation and apoptosis^21^. ADM2 overexpression in the kidney provides a protective effect against renal ischaemia-reperfusion injury possibly by alleviating oxidative stress and consequently suppressing inflammation^22^. ADM2 in the kidney also prevents against IgA nephrology by decreasing oxidative stress and controlling inflammation^23^. Despite these emerging findings regarding the anti-oxidative and anti-inflammatory roles of the ADM family, the effects of exogenous ADM on oxidative stress and inflammatory response in LPS-stimulated Leydig cells have yet to be demonstrate.

To the best of our knowledge, this study is the first to show the anti-oxidant and anti-inflammatory effects of ADM in testicular Leydig cells. We hypothesise that ADM may benefits testicular Leydig cells through its protective effects against oxidative stress and inflammatory response in other cells, tissues and organs. This study explores the protective role and underlying mechanisms of ADM in the attenuation of oxidative stress and inflammatory reaction in rat primary Leydig cells exposed to LPS.

## Materials and Methods

### Reagents

Cell culture dishes, plates, centrifuge tubes and other plastic wares were purchased from BD Biosciences (Lincoln Park, NJ, USA). Rat ADM (1–50) was purchased from Phoenix (Belmont, CA, USA). LPS from *Escherichia coli*, serotype (O127:B8), phosphate buffered saline (PBS), Triton X-100 and the reduced form of nicotinamide adenine dinucleotide phosphate (NADPH) were obtained from Sigma (St. Louis, MO, USA). Dulbecco′s modified Eagle′s medium (DMEM) with the Ham′s F-12 nutrient mixture (at a 1:1 ratio; DMEM-F12), collagenase type IV, Percoll, Trypan blue, bovine serum albumin (BSA), foetal bovine serum (FBS), 3,3′-diaminobenzidine (DAB), 6-dianidino-2-phenylindole dihydrochloride (DAPI) and penicillin/streptomycin were obtained from Gibco (Grand Island, NY, USA). A 5-bromo-2′-deoxyuridine (BrdU) cell proliferation kit was obtained from Millipore (Billerica, MA, USA). Cell counting kit-8 (CCK-8) was purchased from Dojindo Laboratories (Kyushu, Japan). The 2′,7′-dichlorofluorescin diacetate (DCFDA)-cellular ROS detection assay kit was from Abcam (Cambridge, MA, USA). The bicinchoninic acid (BCA) protein assay kit was purchased from Thermo Fisher Scientific (Waltham, MA, USA). The enzyme-linked immunosorbent assay (ELISA) kits for 4-hydroxy-2-nonenal (4-HNE), 8-hydroxy-2-deoxyguanosine (8-OHdG), tumor necrosis factor α (TNF-α), interleukin 1β (IL-1β), transforming growth factor-β1 (TGF-β1), macrophage chemotactic protein 1 (MCP-1) and macrophage migration inhibitory factor (MIF) were purchased from R&D Systems (Minneapolis, MN, USA). DNeasy tissue extraction kit and RNeasy Plus Mini RNA extraction kit were from Qiagen (Valencia, CA, USA). TaqMan gene expression assays and real-time polymerase chain reaction (PCR) master mix were from Applied Biosystems (Foster City, CA, USA). Mouse monoclonal antibody against 8-OHdG and rabbit polyclonal antibodies against 4-HNE, MCP-1 and MIF were obtained from Abcam (Cambridge, MA, USA). Mouse monoclonal antibody against phosphorylated C-Jun N-terminal kinase (JNK), rabbit polyclonal antibodies against JNK, phosphorylated extracellular-signal-regulated kinase (ERK1/2) and IκB-α and rabbit monoclonal antibodies against toll-like receptor (TLR)-2, TLR-4, ERK1/2, phosphorylated p38, p38 and phosphorylated IκB-α were purchased from Cell Signalling Technology (Beverly, MA, USA). Mouse monoclonal antibodies against nuclear factor-kappa B (NF-κB) p65, p50 and β-actin were purchased from Santa Cruz Biotechnology (Santa Cruz, CA, USA). Fluorescein tetramethyl rhodamine isothiocyanate (TRITC)-conjugated and fluorescein isothiocyanate (FITC)-conjugated secondary antibodies (goat anti-mouse or rabbit IgG) were from Molecular Probes (Eugene, OR, USA). All other chemicals used in this study were of analytical grade and obtained from Sigma (St. Louis, MO, USA) unless otherwise stated.

### Animals

Adult Sprague–Dawley rats approximately 90 days old and weighing approximately 400 g were purchased from the Experimental Animal Centre of Wuhan University, China. The experimental protocols used in the study followed the national guidelines and protocols of the National Institutes of Health and were approved by the Local Ethics Committee for the Care and Use of Laboratory Animals of the University of South China. All experimental animals used in the experiments were individually maintained under standard conditions of controlled temperature (22 ± 1 °C), lighting (12 h light:12 h darkness) and humidity (50 ± 10%) with ad libitum diet and water and were used before reaching 120 days.

### Leydig cell isolation, purification and identification

Isolation and purification of rat Leydig cell-enriched preparations were performed similar to that of our previous method^24^. Briefly, eight rats were euthanised with isoflurane followed by cervical dislocation for each isolation event. The testes were dissociated under aseptic conditions and then placed in 50 mL plastic tubes (two testes per tube) filled with DMEM-F12 containing 0.25 mg/mL collagenase and incubated in a thermostatic shaking water bath at constant agitation. After incubation, the enzyme was diluted with collagenase-free DMEM-F12. Tubules were washed again to detach the interstitium and the two cell supernatants were combined. The resulting supernatant containing Leydig cells was filtered through a double layer of 100 µm nylon mesh (Spectrum, Rancho Dominguez, California) and transferred into sterile centrifuge tubes. The cells were collected by centrifugation and the obtained pellet was resuspended in DMEM-F12. Discontinuous Percoll gradients were used to obtain purified Leydig cells from this crude preparation. The Leydig cell suspension was loaded on top of a discontinuous Percoll gradient (5%, 30%, 58% and 70%) and then centrifuged. After centrifugation, most of the purified Leydig cells were observed in the third Percoll gradient. These Leydig cells were carefully collected using a Pasteur pipette, transferred into centrifuge tubes containing DMEM-F12 and then centrifuged. The resulting supernatant was discarded and the residual Percoll was removed by dilution with Percoll buffer solution.

Leydig cell viability was estimated by measuring the percentage of cells that excluded Trypan blue staining method. Briefly, isolated Leydig cells and an equal volume of 0.4% Trypan blue were combined and incubated for 5 min at room temperature. After incubation, an aliquot of cells was loaded into a haemacytometer chamber for cell counting, and the numbers of nonviable (stained) and viable (excluded) cells were counted. Viability was calculated as the percentage of viable cells divided by the total cell count. Leydig cells with at least 95% viability were used for the subsequent experiments.

Leydig cell purity was assessed using histochemical staining for 3-beta-hydroxysteroid dehydrogenase activity as previously described^25^. Briefly, an aliquot of Leydig cell fraction was incubated in 0.1 M PBS at pH 7.4 containing 1 mg/mL nitroblue tetrazolium, 3 mg/mL nicotinamide adenine dinucleotide, 2 mg/mL dehydroepiandrosterone and 1.6 mg/mL nicotinamide for 90 min at 34 °C. Stained cells were washed with PBS once and fixed in 10% formaldehyde for 30 min. Then, the cells were sedimented and washed twice. A drop of resuspended cell suspension was placed on a glass microscope slide. After drying, the percentage of positively stained cells with distinct blue reaction product was counted under an inverted microscope (Olympus, Tokyo, Japan). Leydig cells showed intense staining and were 90% enriched. Depending on the isolation, the yield per isolation from the 16 testes ranged from 24 × 10^6^ to 32 × 10^6^ Leydig cells.

### Cell culture and experimental design

Primary Leydig cells were plated at a density of 1 × 10^6^ cells/well in 6-well plates with a total volume of 2 mL DMEM-F12 containing 3% FBS at a density of 1.25 × 10^5^ cells/well in 24-well plates with a total volume of 1 mL DMEM-F12 containing 3% FBS, or at a density of 1 × 10^4^ cells/well in 96-well plates with a total volume of 200 µL DMEM-F12 containing 3% FBS. The cells were incubated at 37 °C for 24 h under 5% CO_2_ and 95% air. At the end of incubation, the FBS medium was removed, and the cells were incubated with serum-free medium for 1 h before the onset of experimental treatments.

Cells were cultured in 96-well plates with 200 µL serum-free medium in the presence of various doses of ADM (0, 10, 50, 100 and 300 nM) for 12 h to determine the dose-dependent effect of ADM. Cell viability was measured by CCK-8 assay.

To explore the protective effect of ADM on LPS-induced cytotoxicity, cells were incubated in 96-well plates with 200 µL serum-free DMEM containing ADM for 2 h before adding 1 µg/mL LPS. Cells were cultured with serum-free medium in the control group. CCK-8 assay was performed to detect cell viability at 12 h after incubation.

For other experiments, the cells were cultured in 6-well plates with 2 mL serum-free medium or 24-well plates with 1 mL serum-free medium. The cells were divided into four groups, namely, control (cells were cultured in serum-free medium alone), LPS (cells were in serum-free medium containing 1 µg/mL LPS for 12 h), ADM alone (cells were cultured in serum-free medium containing 100 nM ADM for 12 h) and LPS + ADM (cells were cultured in serum-free medium containing 100 nM ADM for 2 h followed by 12 h with 1 µg/mL LPS).

### CCK-8 cell proliferation assay

Cell viability was detected using a CCK-8 assay kit in accordance with the manufacturer′s instructions. Briefly, after implementing the above-described experimental design, the culture medium was removed from each well, and the Leydig cells were washed thrice with 0.1 M PBS. Subsequently, 10 μL CCK-8 solution was added to each well, and the plates were incubated at 37 °C for 2 h. The WST-8(2-(2-methoxy-4-(phenyl)-3-(4-(phenyl)-5-(2,4-sulpho benzene)-2H-tetrazolium monosodium salt) in the reagent can be reduced to orange-yellow formazan by dehydrogenase, which was proportional to the number of viable cells. The absorbance at 450 nm was measured using a microplate reader (Perkin Elmer, Waltham, MA, USA). A standard curve was designed using Leydig cell suspension with different dilution rates to calculate the viable cell numbers in each sample.

### BrdU cell proliferation assay

Cell proliferation was measured using a BrdU cell proliferation kit according to the manufacturer’s instructions. After implementing the above-described experimental design, BrdU (10 μL/well) was added and incubated at 37 °C for 1 h. The medium was removed, and the Leydig cells were washed with PBS. Cell samples were fixed with 4% paraformaldehyde and incubated with 3% H_2_O_2_ in methanol for 15 min to inactivate endogenous peroxidases. Then, the samples were washed with PBS and incubated with 0.1% Triton X-100 on ice for 5 min. After three washes with PBS, cultures were incubated for 2 h with a monoclonal anti-BrdU antibody (1:200). Subsequently, 100 μL/well working solution of TRITC-conjugated goat anti-rabbit secondary antibody (1:1000) was added and incubated at room temperature for 90 min in a dark room. Finally, the cells were rinsed thrice with PBS and were stained with 1 mg/mL DAPI. Immunofluorescent photographs were captured using an inverted microscope (Olympus, Tokyo, Japan) at 200 × magnification. Cell counts were performed on randomly selected areas of the BrdU-stained Leydig cells (visual field at 200 × magnification). We counted the number of BrdU positive cells against the total cell number to calculate the proliferation index (BrdU^+^ cells/total cells).

### Measurement of cellular ROS production

Cellular ROS production was measured using a DCFDA assay kit according to the manufacturer’s instructions. DCFDA as a cell-permeable fluorescent dye was decomposed by cellular esterases to non-fluorescent compound and then oxidised into highly fluorescent dichlorofluorescin (DCF). The amount of intracellular ROS was proportional to the intensity of DCF fluorescence. After Leydig cells were treated in accordance with the above-described experimental design and reached confluence, the culture medium was removed and the cells were incubated with DCFDA (100 µM) at 34 °C for 30 min in the dark. The cells were washed with PBS, resuspended in 200 µL PBS and then fluorescence was measured in a Wallac 1420 microplate reader (Perkin Elmer, Waltham, MA, USA) at an excitation wavelength of 488 nm and emission wavelength of 520 nm. The amount of intracellular ROS was proportional to the intensity of DCF fluorescence, and the fluorescence intensity was recorded directly to indicate the relative amount of ROS. Relative changes of DCF fluorescence were expressed as fold increase over the control cells.

### Immunocytochemistry and immunofluorescent staining

After Leydig cells were treated in accordance with the above-described experimental design, Leydig cells on 6-chamber slides were fixed with 4% paraformaldehyde at 4 °C for 15 min and then permeabilised with 0.2% Triton X-100 in PBS at room temperature for 15 min. The cells were then incubated with primary antibodies against mouse 4-HNE (1:50), 8-OHdG (1:200), MCP-1(1:200), MIF (1:250) and p65 (1:400) at 4 °C overnight. Subsequently, 100 μL/well working solution of goat anti-mouse or rabbit secondary antibodies (1:300) was added and incubated at room temperature for 90 min. After the cells were washed with PBS, they were stained with avidin-biotin-peroxidase complex visualised with DAB for immunocytochemical staining or with DAPI for nuclear counterstaining for immunofluorescent staining. The stained slides were photographed using an inverted microscope (Olympus, Tokyo, Japan) at 200 × magnification. Relative changes of MCP-1 and MIF fluorescence were expressed as fold increase over the control cells.

### Assessment of the antioxidant profile

After Leydig cells were treated according to the above-described experimental design, the medium was removed and 300 µL PBS was added to collect the cells by a cell scraper. The cells were disrupted by an ultrasonic processor, and the homogenate was centrifuged at 5000 × g at 4 °C for 15 min to obtain the supernatant for the enzyme activity assays. The enzyme activities of superoxide dismutase (SOD), catalase (CAT), thioredoxin reductase (TrxR), glutathione peroxidase (GPX), glutathione reductase (GR) and glutathione S-transferase (GST) in the Leydig cells were determined according to the methods as previously described^26,27^. The enzyme activities of SOD, CAT, GPX and GST were expressed as units/mg protein. Activities of TrxR and GR were expressed as μmol/min/mg protein and μmol of NADPH oxidised/min/mg protein. All enzyme activities were normalised against the control group.

### Estimation of TNF-α, IL-1β, TGF-β1, 4-HNE, 8-OHdG, MCP-1 and MIF concentrations

After Leydig cells were treated in accordance with the above-described experimental design, the concentrations of TNF-α, IL-1β and TGF-β1 in the culture medium and 4-HNE, 8-OHdG, MCP-1 and MIF in the Leydig cells were determined with commercial ELISA kits in accordance with the manufacturer′s instructions. The absorbance was measured at 450 nm at a reference wavelength of 570 nm in a microplate reader (Perkin Elmer, Waltham, MA, USA). The protein levels were calculated using a concurrent standard curve. The results of TNF-α, IL-1β and TGF-β1 were expressed as pg/mL. The results of 4-HNE, MCP-1 and MIF were expressed as pg/mg protein and the 8-OHdG levels were expressed as pg/mg DNA. All results were normalised against the control group.

### RNA extraction, reverse transcription and quantitative real-time PCR (RT-qPCR)

After Leydig cells were treated in accordance with the above-described experimental design, the treated cells were dissolved in Trizol reagent and total RNA was extracted with RNeasy Plus Mini RNA extraction kit in accordance with the manufacturer’s instructions. RNA concentration was determined using a spectrophotometer (Evolution 220, Waltham, MA, USA) at 260 nm. Purity was assessed by measuring the ratio of A260/A280. Purified RNA at an A260/A280 ratio between 1.8 and 2.0 was used in this study. The first strand complimentary DNA (cDNA) was synthesised from RNA using reverse transcriptase and a PrimeScript RT reagent kit (Fermentas, Waltham, MA, USA). RT-qPCR was performed in a 7900HT Fast Real-Time PCR system (Applied Biosystems, Foster City, CA, USA) by using SYBR Green chemistry. Each reaction was run in triplicate and performed under standard conditions [25 µL reaction mixture consisting of 0.5 μL 10 mM deoxynucleotide triphosphates, 2.5 μL 10 × buffer (containing Mg^2+^), 1 μL upstream primer (50 μg/mL), 1 μL downstream primer (50 μg/mL), 4 μL cDNA and 1 U Taq enzyme] in 40 cycles consisting of the following steps: initial denaturation at 95 °C for 5 min followed by a set cycle of denaturation at 94 °C for 10 s and different annealing temperatures for each pair of primers (ranging between 53 °C and 62 °C) for 10 s, extension at 72 °C for 28 s and a final elongation at 72 °C for 5 min. Primer sequences of the targeted genes used for this study were as follows: TNF-α (5′-AAATGGGCTCCCTCTCATCAGTTC-3′, forward; 5′-TCTGCTTGGTGGTTTG CTACGAC-3′, reverse; NM004628.4), IL-1β (5′-CATTGTGGCTGTGGAGAAG-3′, forward; 5′-ATCATCCCACGAGTCACAGA-3′, reverse; NM005529.6), TGF-β1 (5′-CCCAGCATCTGCAAAGCTC-3′, forward; 5′-GTCAATGTACAGCTGCCGCA-3′, reverse; NM000660.6), TLR-2 (5′-TCGAGAAGAGCCACAAAACC-3′, forward; 5′- CGAAAATGGGAGAAGTCCAG-3′, reverse; NM001318796.1), TLR-4 (5′- ACAAAAGCCCAGAACGCTAA-3′, forward; 5′-TGCACAGAGAGCAGTTTTTCA-3′, reverse; NM015534.5) and β-actin (5′-CGTTGACATCCGTAAAGAC-3′, forward; 5′-TGGAAGGTGGACAGTGAG-3′, reverse; NM001199954.1). The generation of specific PCR products subjected to melting curve analysis for each primer set revealed only one peak for each product. All the gene expression levels were normalised for expression of the housekeeping gene, β-actin, and expressed as the fold ratio compared with the control group.

### Western blot analysis

After Leydig cells were treated according to the above-described experimental design, the entire cell lysates and cytoplasmic and nuclear extracts were harvested from Leydig cell monolayers. The total protein concentrations of the entire cell and cytoplasmic and nuclear extracts were measured with a BCA assay kit using BSA as the standard. After adjusting for equal amounts of total protein, protein mixtures were separated by sodium dodecyl sulfate-polyacrylamide gel electrophoresis and transferred to polyvinylidene difluoride membranes. After the transfer, non-specific binding sites of the membranes were blocked for 1 h at room temperature in PBS at pH 7.4 containing 5% (wt/vol) nonfat dry milk and subsequently incubated with primary antibodies against TLR-2 (1:1000), TLR-4 (1:1000), phosphorylated JNK (1:2000), JNK (1:1000), phosphorylated ERK1/2 (1:1000), ERK1/2 (1:1000), phosphorylated p38 (1:1000) and p38 (1:1000), phosphorylated IκB-α(1:1000), IκB-α (1:1000), NF-κB p65 (1:1000) and p50 (1:1000) at 4 °C overnight. The membranes were probed with an anti-β-actin antibody (1:1000) to control for protein loading. Then, the membranes were incubated for 2 h at room temperature with horseradish peroxidase-conjugated secondary antibodies (1:1000). The results were scanned using a gel imaging system (UVP Company, Upland, CA, USA). Densitometry measurements were performed with Image Lab software (BioRad Laboratories, Hercules, CA, USA). The band intensities were semiquantified by densitometry using Quantity-One software (BioRad Laboratories, Hercules, CA, USA). Relative protein expression was normalised to β-actin and compared with the control group.

### Statistical analysis

Data were expressed as mean ± stand error of the mean (SEM) of the average of the three wells in each of the five experiments. Data were analysed using SPSS version 19.0 (SPSS Inc., Chicago, IL, USA). Significant differences among the mean values of multiple groups were evaluated with one-way ANOVA followed by Student-Newman-Keuls’ method. A two-sided *P* value < 0.05 was considered statistically significant.

## Results

**Figure 1:**
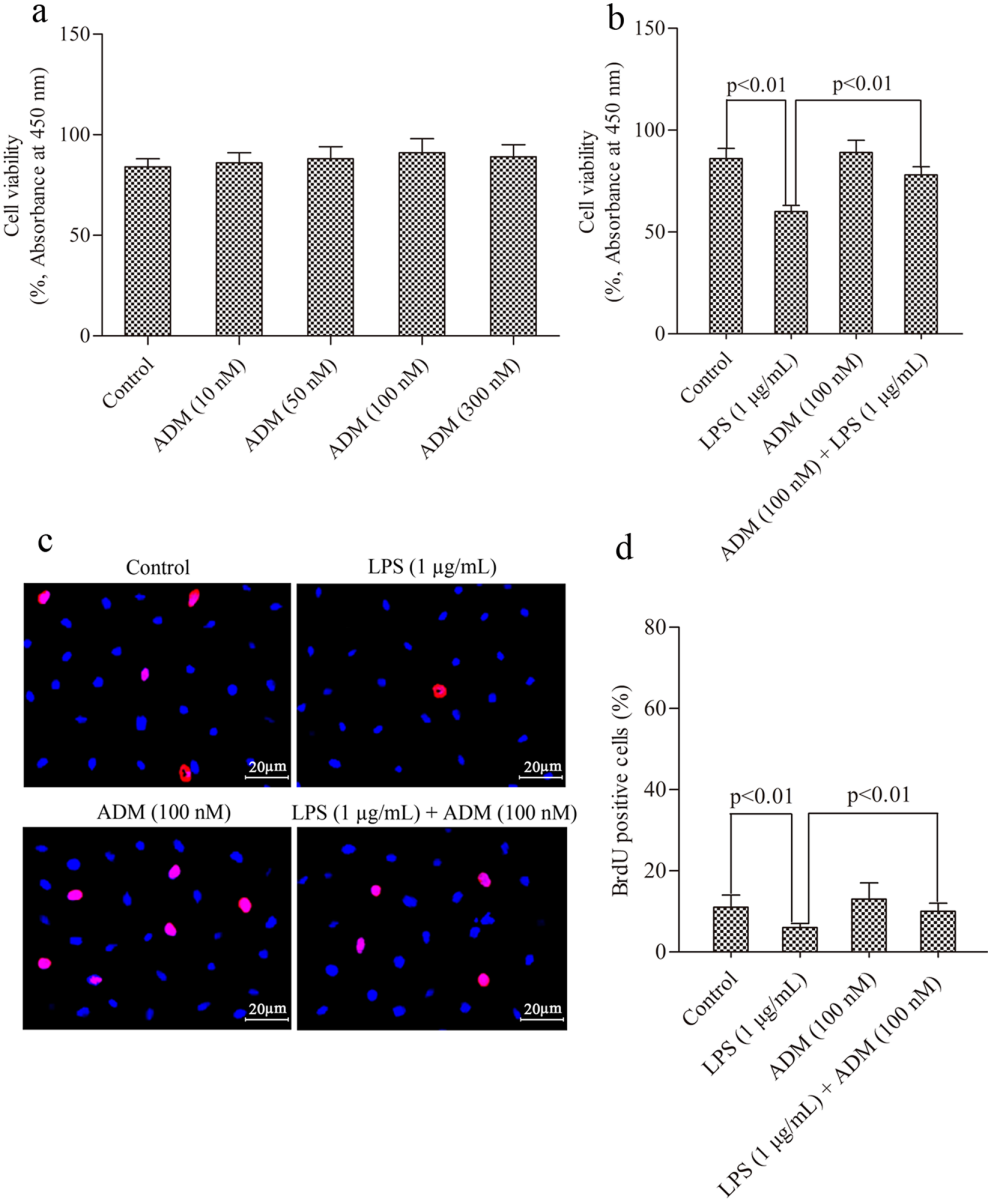
Effect of ADM on cell viability and the LPS-induced damage of Leydig cells and cell proliferation. (**a**) Dose-response effect of ADM (0, 10, 50, 100, or 300 nM) on the viability of Leydig cells after a 12-h treatment. (**b**,**c**) Protective effect of ADM (100 nM) on cell damage induced by LPS and on cell proliferation suppressed by LPS (1 µg/mL). Scale bar: 20 µm. (**d**) Statistical analysis of percentage of BrdU positive cells. Data were obtained from five independent experiments performed in triplicate and expressed as mean ± SEM.

The effect of ADM on cell viability, the LPS-induced damage of Leydig cells and cell proliferation are shown in Fig. 1. No significant differences were observed in the absorbance among the five treated groups at different ADM concentrations. However, the group with 100 nM ADM exerted a relatively strong effect (Fig. 1a). LPS significantly reduced cell viability and suppressed cell proliferation (*P* < 0.01), which were significantly reversed by the addition of 100 nM ADM (*P* < 0.01) (Fig. 1b,c and d).

**Figure 2:**
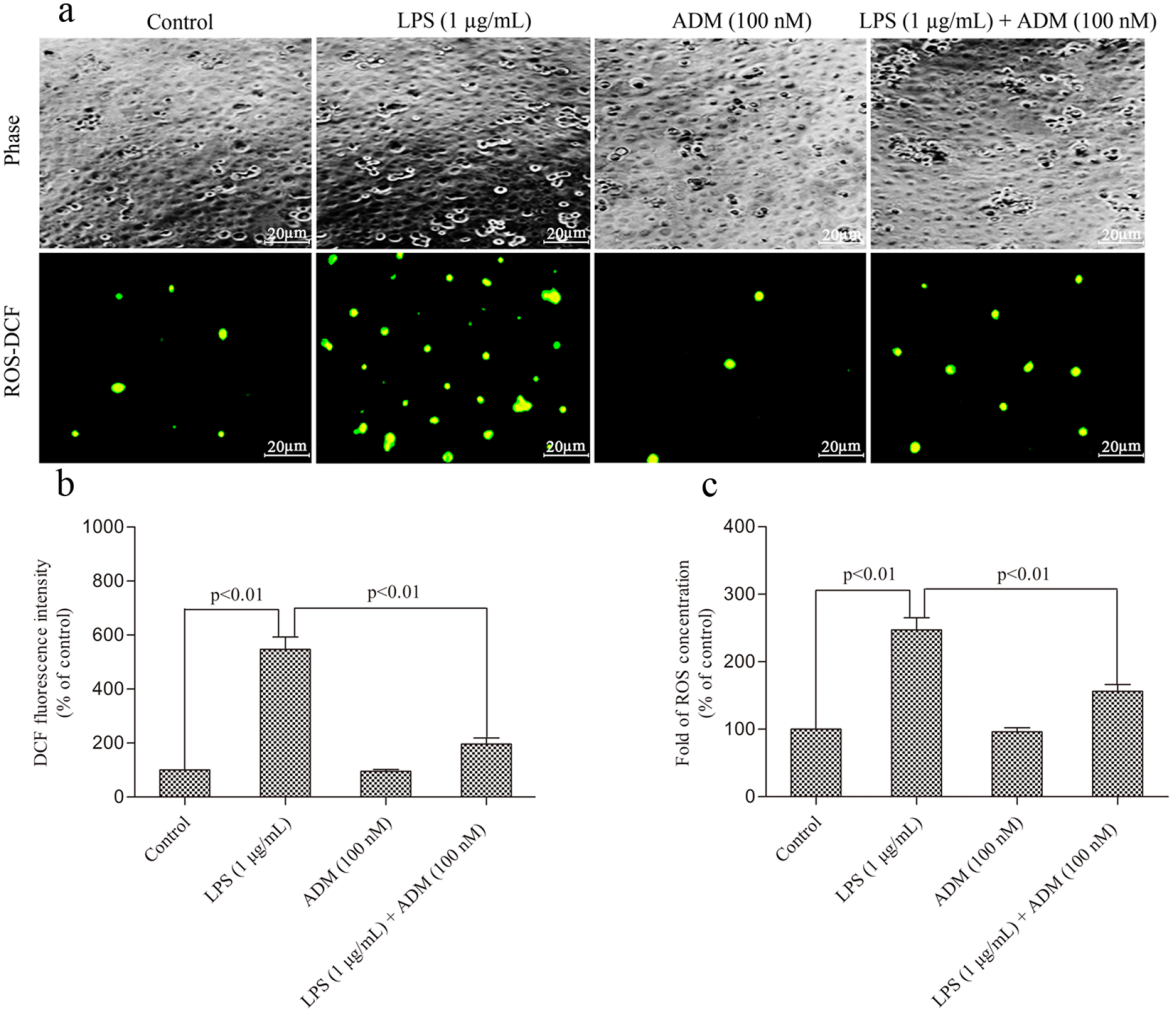
ADM blocked ROS overproduction in the primary culture of Leydig cells exposed to LPS. (**a**) Representative images showing positive cells of ROS-DCF fluorescence in different treated groups. The phase images showing equal cell density in the four groups. Scale bar: 20 µm. (**b**) ROS production by Leydig cells in different treated groups measured by DCF fluorescence intensity. (**c**) Graph displayed increase of relative folds of ROS production normalised by the control group. Data were obtained from five independent experiments performed in triplicate and expressed as mean ± SEM.

As shown in Fig. 2, ADM pretreatment significantly reduced ROS levels. LPS significantly increased the ROS fluorescence-positive cells when compared with the control group (*P* < 0.01), which was significantly reduced when ADM was added prior to LPS (*P* < 0.01) (Fig. 2a). Compared with the control group, the DCF fluorescence intensity was significantly stimulated when the cells were exposed to LPS (*P* < 0.01), which was significantly decreased by ADM supplementation (*P* < 0.01) (Fig. 2b). ROS concentration further showed that LPS significantly increased ROS production in Leydig cells when compared with the control group (*P* < 0.01). This phenomenon was significantly ameliorated by ADM addition (*P* < 0.01) (Fig. 2c).

**Figure 3:**
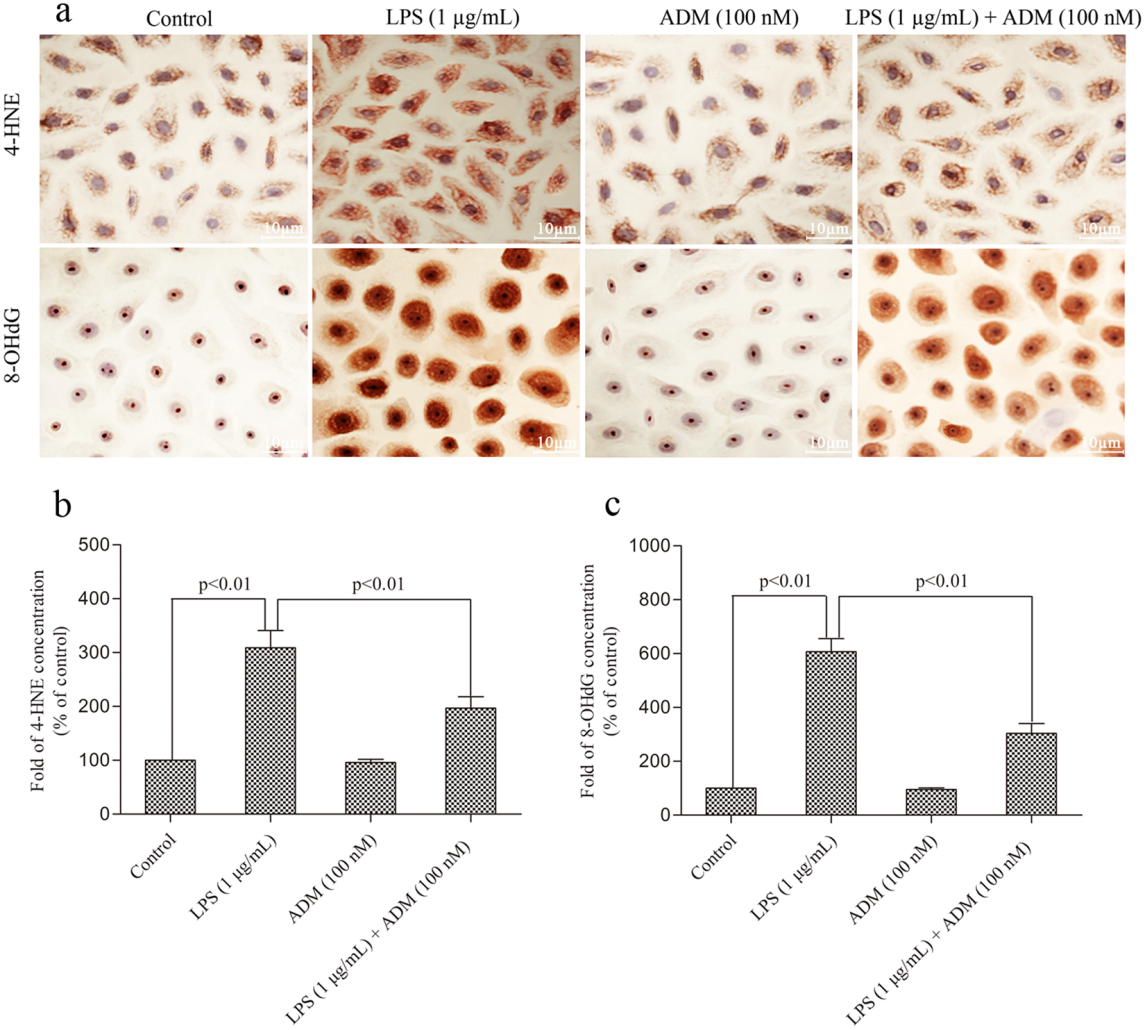
Representative images showing the effect of ADM on the LPS-induced expression levels and production of 4-HNE and 8-OHdG. (**a**) Representative immunocytochemical images showing increased immunoreactivities of 4-HNE and 8-OHdG in primary Leydig cells in different treated groups. Scale bar: 10 µm. (**b**) Concentrations of 4-HNE and 8-OHdG in different treated groups. Data were obtained from five independent experiments performed in triplicate and expressed as mean ± SEM.

Figure 3 shows the effect of ADM on the LPS-induced expression levels and the production of 4-HNE and 8-OHdG. Stimulation with LPS significantly increased the expression levels and production of 4-HNE and 8-OHdG (*P* < 0.01). When ADM was added prior to LPS, the expression levels of 4-HNE and 8-OHdG and the concentrations of 4-HNE and 8-OHdG significantly reduced (*P* < 0.01).

**Figure 4:**
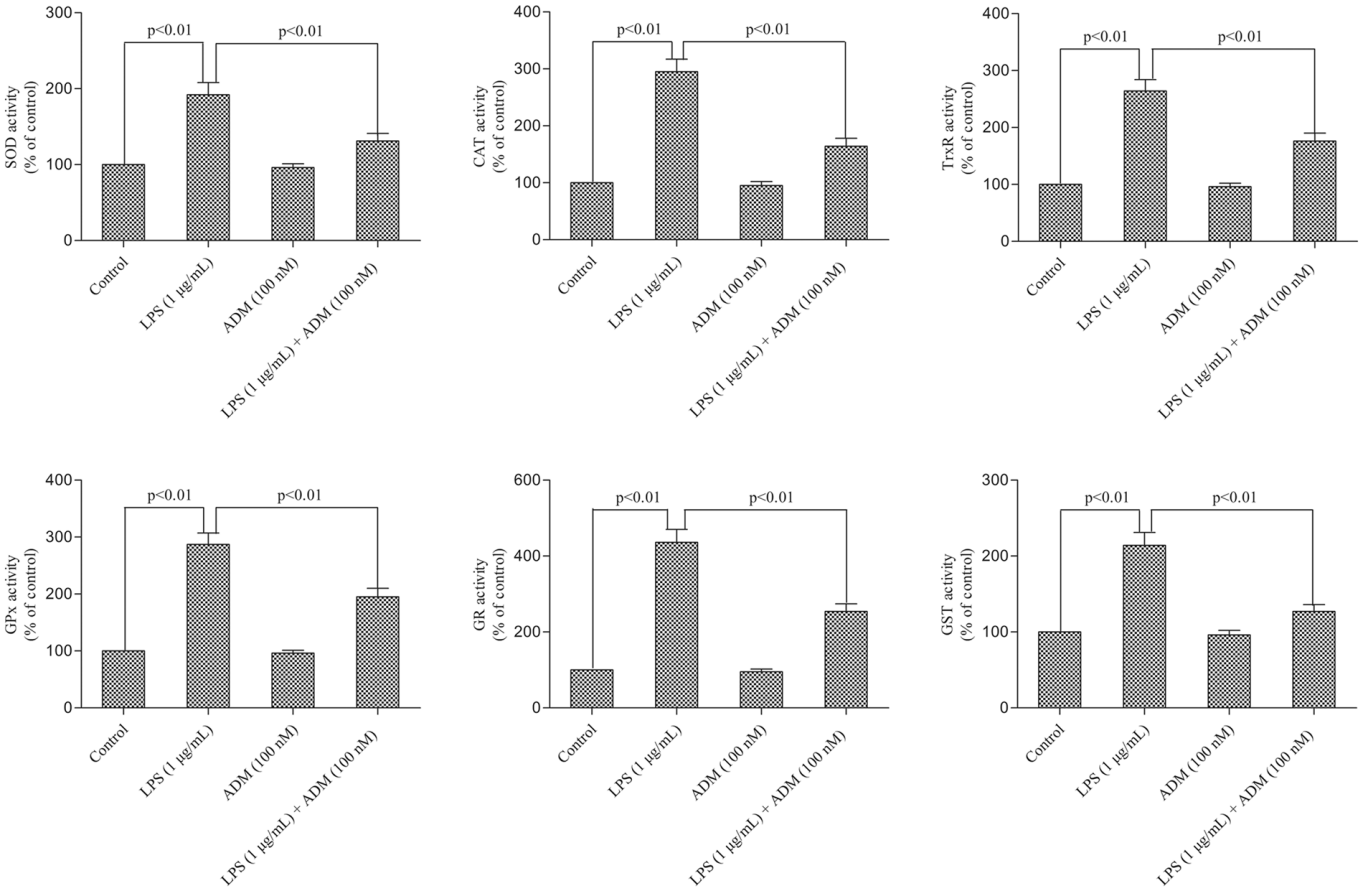
Effect of ADM on the enzymatic activities of SOD, CAT, TrxR, GPX, GR and GST. Primary Leydig cells were treated with ADM (100 nM) for 2 h and then switched to the culture medium in the presence of LPS (1 µg/mL) for 12 h to evaluate the enzymatic activities. Data were obtained from five independent experiments performed in triplicate and expressed as mean ± SEM.

The effect of ADM on LPS-induced production of antioxidant enzymatic activities is depicted in Fig. 4. Stimulation with LPS caused significant increases in the enzymatic activities of SOD, CAT, TrxR, GPX, GR and GST compared with the control group (*P* < 0.01). After ADM pretreatment, the enzymatic activities of SOD, CAT, TrxR, GPX, GR and GST significantly reduced (*P* < 0.01).

**Figure 5:**
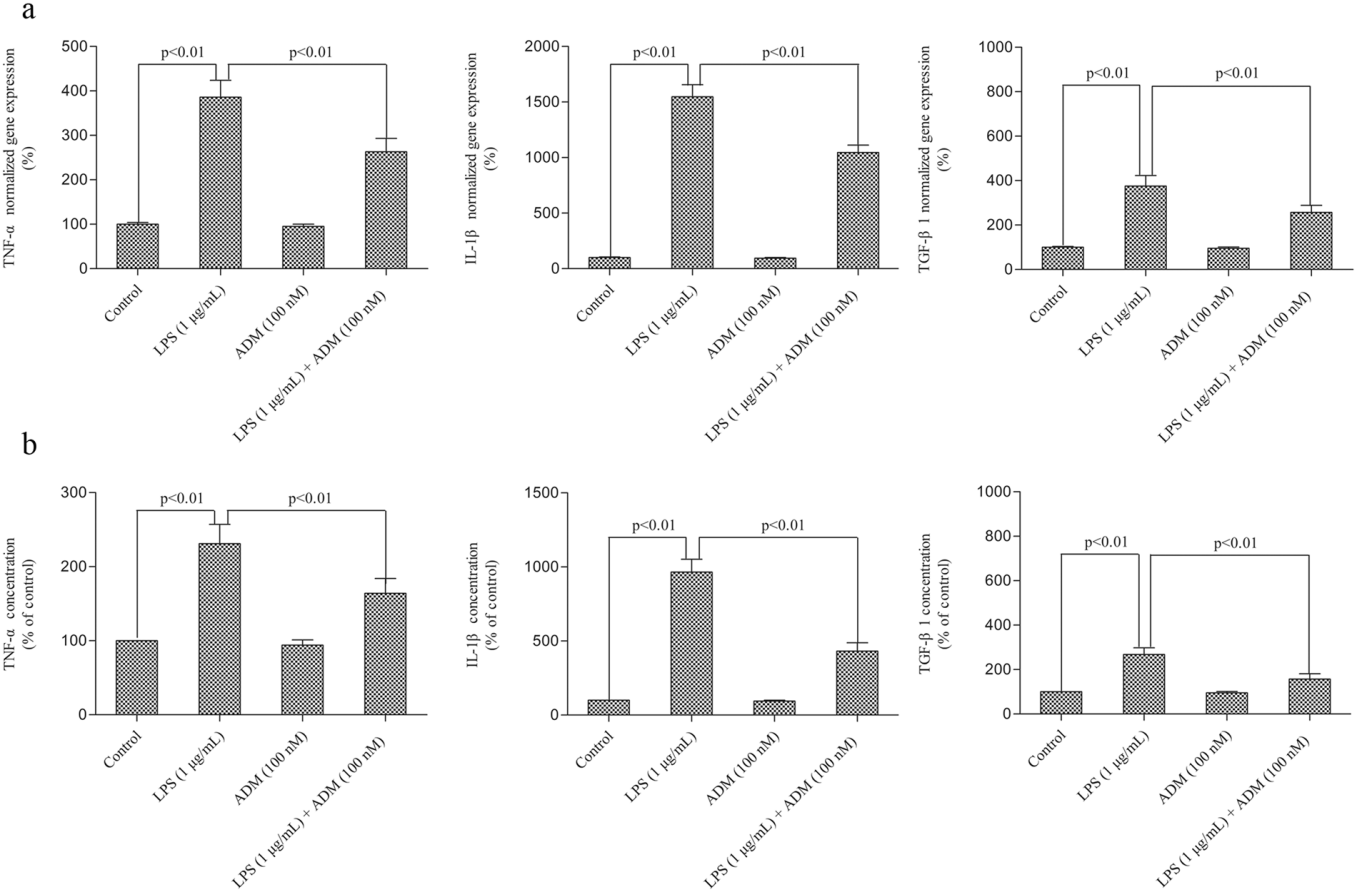
Effect of ADM on the LPS-induced expression levels and production of TNF-α, IL-1β and TGF-β1 in primary Leydig cells. (**a**,**b**) Primary Leydig cells were treated with ADM (100 nM) for 2 h and then switched to the culture medium in the presence of LPS (1 µg/mL) for 12 h to evaluate the mRNA levels by RT-qPCR and measured the protein levels by ELISA. The normalised levels of gene expression are expressed as ratios of the copy number of the mRNA and that of β-actin cDNA. Data were obtained from five independent experiments performed in triplicate and expressed as mean ± SEM.

Figure 5 shows the effect of ADM on the LPS-induced gene expression levels and the production of TNF-α, IL-1β and TGF-β1. Stimulation with LPS significantly increased the gene expression levels of TNF-α, IL-1β and TGF-β1 (*P* < 0.01), as well as the production of TNF-α, IL-1β and TGF-β1 in the supernatant (*P* < 0.01). When ADM was added prior to LPS, the gene expression levels of TNF-α, IL-1β and TGF-β1 and the concentrations of TNF-α, IL-1β and TGF-β1 significantly reduced (*P* < 0.01).

**Figure 6:**
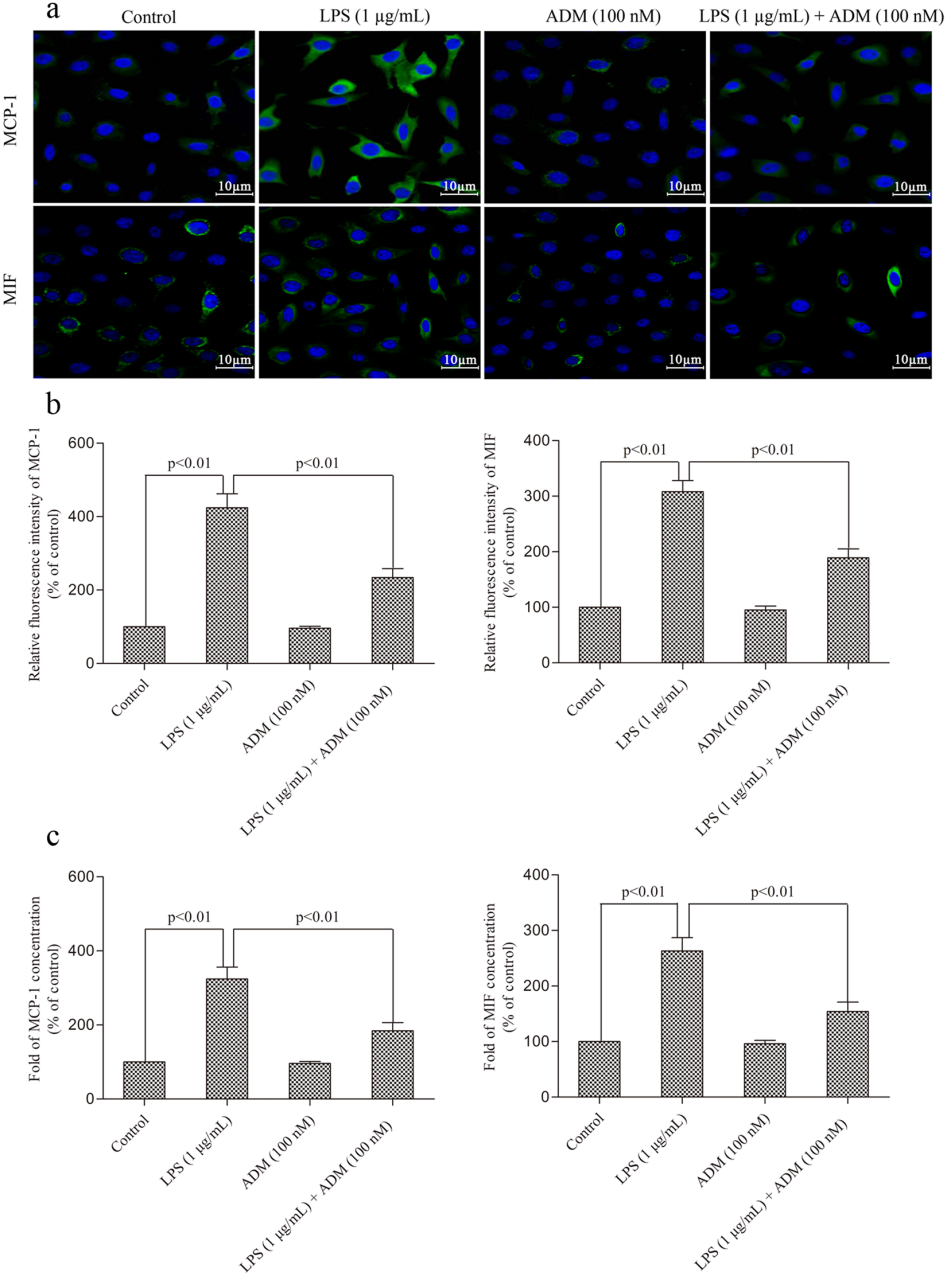
Representative images showing the effect of ADM on the LPS-induced expression levels and production of MCP-1 and MIF. (**a**) Representative immunofluorescent images showing increased immunoreactivities of MCP-1 and MIF in primary Leydig cells in different treated groups. Scale bar: 10 µm. (**b**) Fluorescence intensity of MCP-1 and MIF by Leydig cells in different treated groups. (**c**) Graph displayed increases of relative fold of MCP-1 and MIF production normalised by the control group. Data were obtained from five independent experiments performed in triplicate and expressed as mean ± SEM.

Figure 6 shows the effect of ADM on the LPS-induced expression levels and the production of MCP-1 and MIF. LPS caused significant increases in the expression levels and production of MCP-1 and MIF compared with the control group (*P* < 0.01). When ADM was added prior to LPS, the expression levels and concentrations of MCP-1 and MIF significantly reduced (*P* < 0.01).

**Figure 7:**
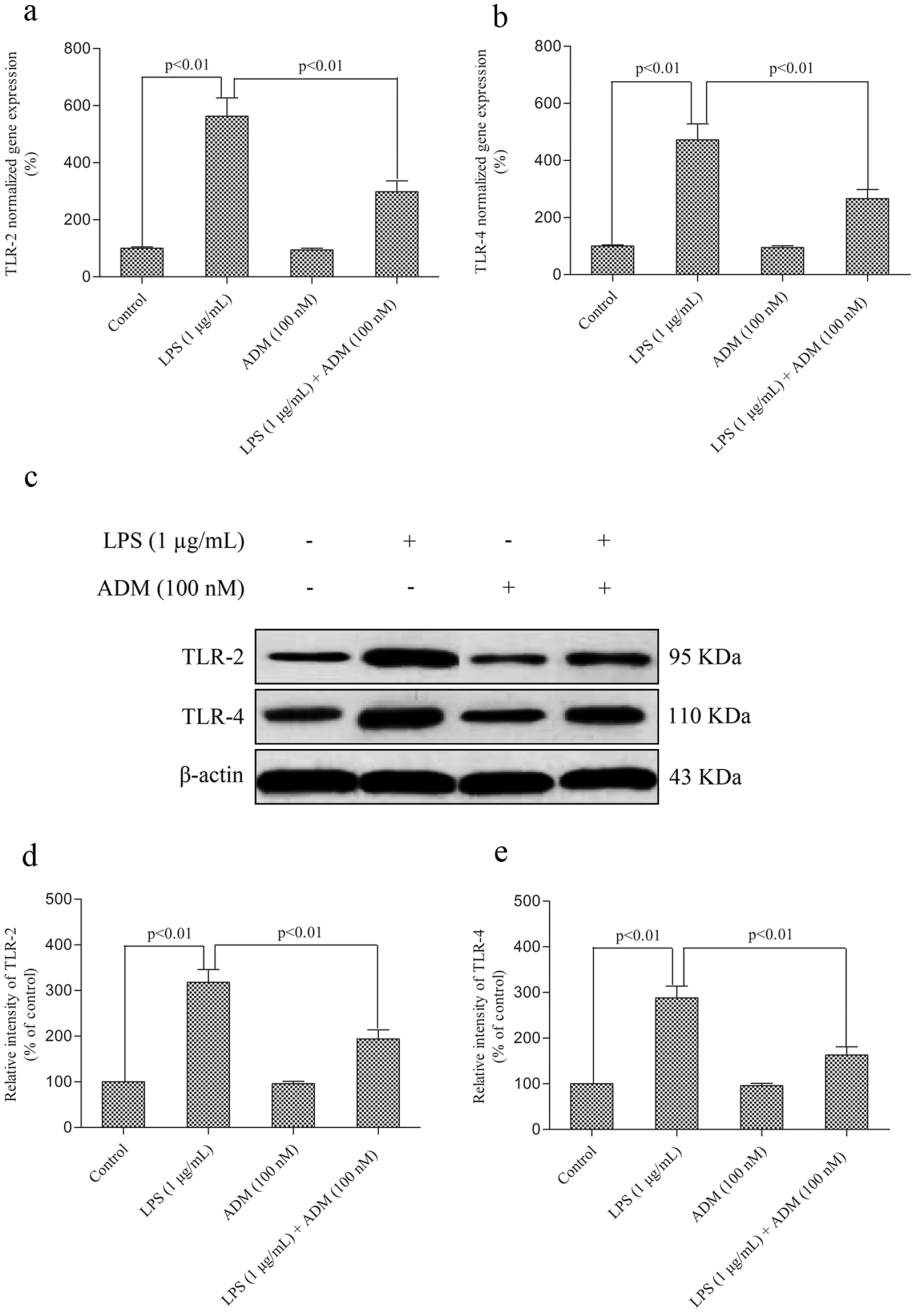
Effect of ADM on the LPS-induced gene expression levels and protein production of TLR2 and TLR4 in primary Leydig cells. (**a**,**b**) Primary Leydig cells were treated with ADM (100 nM) for 2 h and then switched to the culture medium in the presence of LPS (1 µg/mL) for 12 h to evaluate the mRNA levels by RT-qPCR. (**c**) Representative images of Western blot with anti-TLR2 and TLR4 antibodies after primary Leydig cells were treated with ADM (100 nM) for 2 h and then switched to the culture medium in the presence of LPS (1 µg/mL) for 12 h. (**d**,**e**) Statistical analysis of Western blot results. The normalised levels of gene expression are expressed as ratios of the copy number of the mRNA and that of β-actin cDNA. β-actin was used as internal reference. Data were obtained from five independent experiments performed in triplicate and expressed as mean ± SEM.

The effect of ADM on LPS-induced gene expression levels and the production of TLR2 and TLR4 is shown in Fig. 7. Stimulation with LPS led to significant increases in the gene expression levels and protein levels of TLR2 and TLR4 (*P* < 0.01). When ADM was added prior to LPS, the gene expression levels and protein levels of TLR2 and TLR4 significantly decreased (*P* < 0.01).

**Figure 8:**
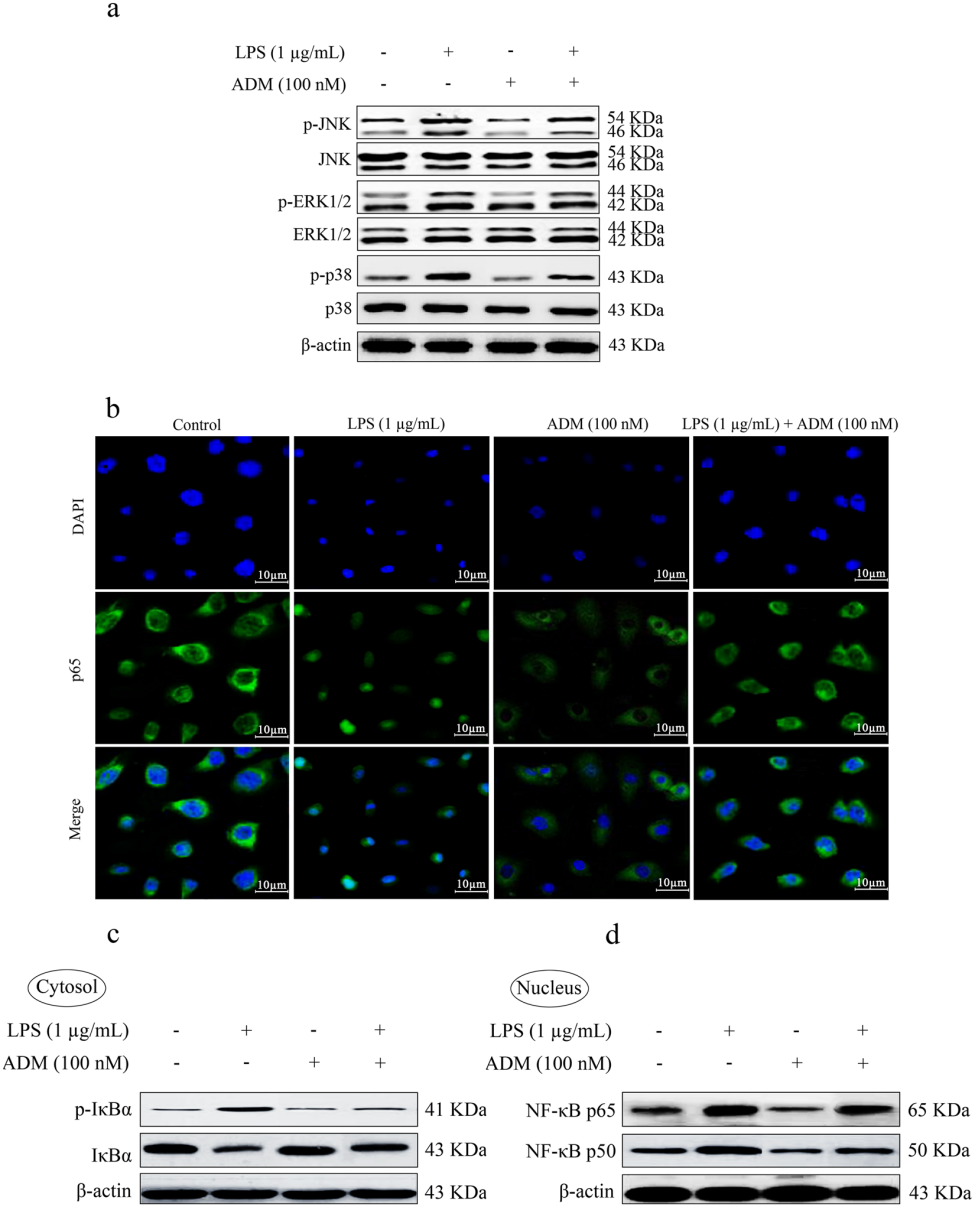
Effect of ADM on the LPS-induced phosphorylation of JNK, ERK1/2 and p38 and nuclear translocation of p65. Primary Leydig cells were treated with ADM (100 nM) for 2 h before a 12-h treatment with LPS (1 µg/mL). (**a**) Phosphorylation of JNK, ERK1/2 and p38 in primary Leydig cells in different treated groups. (**b**) Nuclear translocation of p65 and the p-IκBα in the cytoplasm and p-p65 and p-p50 in the nucleus in primary Leydig cells in different treated groups. Scale bar: 10 µm. (**c**,**d**) Representative images of Western blot with anti-p-IκBα and IκBα antibodies in cytosol and anti-p-p65 and p-p50 antibodies in nucleus after primary Leydig cells were treated with ADM (100 nM) for 2 h and then switched to the culture medium in the presence of LPS (1 µg/mL) for 12 h. β-actin was used as internal reference. Data were obtained from five independent experiments performed in triplicate and expressed as mean ± SEM.

Figure 8 shows the effect of ADM on the LPS-induced phosphorylation of JNK, ERK1/2 and p38 and nuclear translocation of p65. In protein extracts from primary Leydig cells, elevated levels of LPS-induced phosphorylation of JNK, ERK1/2 and p38 were observed compared with the control group without altering total JNK, ERK1/2 and p38 levels (*P* < 0.01). The increased protein levels of p-JNK, p-ERK1/2 and p-p38 were significantly reduced by ADM pretreatment (*P* < 0.01). Increased levels of LPS-induced phosphorylation of IκBα, p65 and p50 were also observed compared with the control group (*P* < 0.01). The protein levels of p-IκBα, p-p65 and p-p50 were significantly inhibited by ADM pretreatment (*P* < 0.01).

## Discussion

ADM can reduce the inflammatory response by downregulating the production of inflammatory mediators, including cytokines, chemokines and free radicals^28^. By reducing ROS production via a protein kinase A-dependent pathway, ADM might possess the endogenous antioxidant potential to protect against ROS-induced podocyte injury^29^. ADM exerts anti-inflammatory and anti-bacterial effects by inducing the downregulation of inflammatory cytokines in cultured cells and attenuating inflammatory processes in a variety of different colitis models^30^. By reversing the deteriorations in mortality and inflammatory responses, ADM regulates systemic inflammation and protects against liver damage in LPS-induced endotoxemia^16^. Continuous infusion of ADM ameliorates the LPS-induced acute lung injury in rats by inhibiting inflammation^31^.

In the present study, the effect of ADM on the cell viability of Leydig cells and the protective effect of ADM on the LPS-induced damage of Leydig cells were relatively the strongest when the ADM concentration was 100 nM. The protective effect of 100 nM ADM was further confirmed by reversing the LPS-induced inhibition of Leydig cell proliferation. These findings are consistent with those of our previous report^24^.

ADM in the seminal secretion may modify the inflammatory responses, play an anti-oxidative role and increase leukocyte and macrophage infiltration in the uterus^32^. In our study, ADM pretreatment significantly reduced the LPS-induced production of ROS. Thus, ADM possibly exerts an anti-oxidative role in Leydig cells by inhibiting LPS-induced excessive oxidative stress. Lipid peroxidation of the cell membrane is one of the major consequences of ROS overproduction, leading to the production of conjugated diene hydroperoxides and unstable substances that disintegrate into various aldehydes, such as 4-HNE, one of the biomarkers for lipid oxidative damage^33^. In nuclear and mitochondrial DNA, 8-OHdG is a predominant form of free radical-induced oxidative lesions and therefore widely used as a biomarker for oxidative stress^34^. In the present study, ADM pretreatment significantly reduced the LPS-induced production of 4-HNE and 8-OHdG. Hence, ADM may act as an anti-oxidative agent by inhibiting LPS-induced lipid oxidative damage and oxidative DNA damage.

Free radical generation, whether from endogenous or exogenous sources, occurs continuously within cells as a consequence of common metabolic processes^35^. Free radicals can lead to oxidative stress, a harmful process that causes serious damages to all biomolecules in cells, thus impairing cell functions and even resulting in cell death and diseased states^36^. Enzymatic antioxidants, such as SOD, CAT, TrxR, GPX, GR and GST are the natural defence system against free radical-mediated tissue damage in several organs, including the testis^37,38^. Our data suggested that the increased activities of enzymatic antioxidants are partially due to excessive oxidative stress and associated damage in LPS-exposed Leydig cells. The elevated activities of these enzymes, which are responsible for the detoxification of free radical generation, may be involved in the mechanisms counteracting an overactivation in oxidative stress. This condition might be a protective mechanism for ADM in LPS-exposed Leydig cells.

LPS can induce the production of inflammatory factors, such as TNF-α and IL-1β, and subsequently suppress steroidogenesis by Leydig cells^39^. In an *in vivo* model of LPS injection to induce inflammatory response, the expression levels of TGF-β1 and MIF in the testis are not dependent on the presence of intact Leydig cells but are under direct testosterone control^40^. The ability of the Sendai virus to induce chemokine production in Leydig cells, such as MCP-1, may therefore increase leukocyte recruitment to the infection sites^41^. In the present study, ADM pretreatment significantly reduced the overexpression of LPS-induced gene and the overproduction of TNF-α, IL-1β, TGF-β1, MCP-1 and MIF. These findings suggested that ADM may attenuate LPS-induced uncontrolled inflammation in Leydig cells by reducing the production of some inflammatory cytokines and inhibiting the recruitment of leukocytes to infectious sites. This behaviour indicates the therapeutic potential of ADM on LPS-induced inflammatory conditions of Leydig cells.

TLR-3 and TLR-4 can be activated by their agonists, such as LPS in Leydig cells, and subsequently induce the production of inflammatory factors^39^. Mumps virus can induce innate immune responses and suppress testosterone synthesis in mouse Leydig cells through TLR-2, resulting in the production of proinflammatory cytokines and chemokines^42^. Our present study is the first work to reveal that ADM pretreatment decreased the gene expression and protein levels of TLR-2 and TLR-4 in LPS-induced inflammation in Leydig cells. Our work demonstrated that ADM may protect Leydig cells from blocking LPS-induced inflammation by inhibiting the binding of LPS to its receptors. This effect may be a highly important anti-inflammatory mechanism for ADM in LPS-exposed Leydig cells.

We further investigated whether the MAPK/NF-κB signalling pathways are involved in the anti-oxidative and anti-inflammatory activities of ADM in response to LPS in Leydig cells. Several studies have reported that the MAPK super family is one such signalling cascade, which includes ERK1/2, JNK and p-38 kinases that are involved in the transduction of signals from the cell membrane to the nucleus^43^. We focused on the MAPK/NF-κB pathways because the activation of MAPK and NF-κB signalling is implicated in stimulating oxidative stress and inflammatory response. The oxidative stress caused by ROS can activate the MAPK signalling pathways, which further activate several inflammatory cytokines^44^. Our results demonstrated that ADM pretreatment decreased the activity of LPS, an effect that attenuated the phosphorylation of JNK, ERK and p38 and verified the proinflammatory nature of the MAPK signalling pathways in Leydig cells. These results suggested that the suppression of JNK, ERK and p38 phosphorylation might be implicated in the inhibition of oxidative stress and proinflammatory mediators and cytokines in LPS-stimulated Leydig cells. ADM may exert its anti-inflammatory effect on rat Leydig cells by inhibiting MAPK phosphorylation. NF-κB is a nuclear transcription factor that can be activated by various cytokines and stimuli, which finally regulate the expression of a large number of genes that are necessary to regulate inflammation^45^. LPS can activate numerous intracellular signalling pathways, such as MAPKs, which may converge on NF-κB^46^. Excessive ROS induced by LPS intermediates can lead to the increased production of cytokines, such as IL-1β, IL-6 and TNF-α^6^. Our previous study demonstrated that excessive ROS induced by IL-1β can also function as signalling messengers to NF-κB and ultimately lead to increased production of inflammatory cytokines, such as NO and prostaglandin E2 in Leydig cells. However, ADM can protect Leydig cells against inflammatory responses by inhibiting NF-κB activity^47^. In the current study, ADM inhibited the LPS-induced phosphorylation and degradation of IκB-a and the nuclear translocation of NF-κB p65 and p50 subunits. Therefore, ADM may inhibit oxidative stress and prevent the production of proinflammatory mediators and cytokines through NF-κB inactivation by reducing NF-κB p65 nuclear translocation and IκB-α phosphorylation and degradation. These findings indicated that ADM could exert anti-oxidative and anti-inflammatory activities by downregulating the MAPK signalling pathway and NF-κB activation in Leydig cells exposed to LPS, which might represent a target of ADM on oxidative stress and inflammatory response of LPS-induced rat Leydig cells.

Our study is characterized by several limitations. Firstly, results were obtained from an *in vitro* cell model. As such, *in vivo* studies and clinical trials should be performed to verify whether an equivalent effect would be observed. Secondly, all our data were acquired through ADM pretreatment experiments, which are necessary to allow the protective role of the compound. Thirdly, the endogenous ADM from Leydig cells may influence the involvement of exogenous ADM, and this effect may overestimate the protective role of ADM. Future studies should also be conducted to elucidate the precise mechanism of ADM regulation in testosterone production and determine the associated enzymes in Leydig cells.

In conclusion, ADM inhibited LPS-induced oxidative stress and inflammation via a mechanism possibly associated with the MAPK/NF-κB signalling pathways. Our results could be extrapolated to idiopathic male infertility involving a remarkable proinflammatory microenvironment that can induce excessive oxidative stress in the testis. Our study also provided a partial molecular explanation for the anti-inflammatory properties of ADM.

### Ethical approval

This study followed the national guidelines and protocols of the National Institutes of Health and was approved by the Local Ethics Committee for the Care and Use of Laboratory Animals of University of South China.

## Electronic supplementary material

Dataset 7 and 8

## References

[1] Chen Q, Deng T, Han D (2016). Testicular immunoregulation and spermatogenesis. Seminars in cell & developmental biology..

[2] Tremblay JJ (2015). Molecular regulation of steroidogenesis in endocrine Leydig cells. Steroids..

[3] Schagdarsurengin U, Western P, Steger K, Meinhardt A (2016). Developmental origins of male subfertility: role of infection, inflammation, and environmental factors. Seminars in Immunopathology..

[4] Azenabor A, Ekun AO, Akinloye O (2015). Impact of Inflammation on Male Reproductive Tract. Journal of reproduction & infertility..

[5] Inoue T (2015). Endogenous interleukin 18 regulates testicular germ cell apoptosis during endotoxemia. Reproduction (Cambridge, England)..

[6] Li L (2013). Intermedin attenuates LPS-induced inflammation in the rat testis. PloS one..

[7] Sadasivam M, Ramatchandirin B, Ayyanar A, Prahalathan C (2014). Bacterial lipopolysaccharide differently modulates steroidogenic enzymes gene expressions in the brain and testis in rats. Neuroscience research..

[8] Wang H (2014). Maternal LPS exposure during pregnancy impairs testicular development, steroidogenesis and spermatogenesis in male offspring. PloS one..

[9] Ramatchandirin, B., Sadasivam, M., Kannan, A. & Prahalathan, C. Sirtuin 4 Regulates Lipopolysaccharide Mediated Leydig Cell Dysfunction. Journal of cellular biochemistry **117** (2016). 10.1002/jcb.25374 26365714

[10] Allen JA, Diemer T, Janus P, Hales KH, Hales DB (2004). Bacterial endotoxin lipopolysaccharide and reactive oxygen species inhibit Leydig cell steroidogenesis via perturbation of mitochondria. Endocrine..

[11] Reddy MM (2006). Bacterial lipopolysaccharide-induced oxidative stress in the impairment of steroidogenesis and spermatogenesis in rats. Reproductive toxicology (Elmsford, N.Y.)..

[12] Metukuri MR, Reddy CM, Reddy PR, Reddanna P (2010). Bacterial LPS-mediated acute inflammation-induced spermatogenic failure in rats: role of stress response proteins and mitochondrial dysfunction. Inflammation..

[13] Itoh T (2007). Adrenomedullin ameliorates lipopolysaccharide-induced acute lung injury in rats. American journal of physiology. Lung cellular and molecular physiology..

[14] Kato J, Kitamura K (2015). Bench-to-bedside pharmacology of adrenomedullin. European journal of pharmacology..

[15] Kubo K (2014). Biological properties of adrenomedullin conjugated with polyethylene glycol. Peptides..

[16] Saito R (2012). Function of adrenomedullin in inflammatory response of liver against LPS-induced endotoxemia. APMIS: acta pathologica, microbiologica, et immunologica Scandinavica..

[17] Oyar EO (2011). Adrenomedullin attenuates aortic cross-clamping-induced myocardial injury in rats. American journal of surgery..

[18] Talero E (2012). Anti-inflammatory effects of adrenomedullin on acute lung injury induced by Carrageenan in mice. Mediators of inflammation..

[19] Tao W, Shu YS, Miao QB, Zhu YB (2012). Attenuation of hyperoxia-induced lung injury in rats by adrenomedullin. Inflammation..

[20] Zhang S, Patel A, Moorthy B, Shivanna B (2015). Adrenomedullin deficiency potentiates hyperoxic injury in fetal human pulmonary microvascular endothelial cells. Biochemical and biophysical research communications..

[21] Li H (2013). Intermedin protects against myocardial ischemia-reperfusion injury in diabetic rats. Cardiovascular diabetology..

[22] Qiao X (2013). Intermedin protects against renal ischemia-reperfusion injury by inhibition of oxidative stress. American journal of physiology. Renal physiology..

[23] Park WJ (2015). Analysis of cytokine production in a newly developed canine tracheal epithelial cell line infected with H3N2 canine influenza virus. Archives of virology..

[24] Zhou PH, Hu W, Zhang XB, Wang W, Zhang LJ (2016). Protective Effect of Adrenomedullin on Rat Leydig Cells from Lipopolysaccharide-Induced Inflammation and Apoptosis via the PI3K/Akt Signaling Pathway ADM on Rat Leydig Cells from Inflammation and Apoptosis. Mediators of inflammation..

[25] Jiang X (2015). Effects of treatment with Astragalus Membranaceus on function of rat leydig cells. BMC complementary and alternative medicine..

[26] Murugesan P, Muthusamy T, Balasubramanian K, Arunakaran J (2008). Polychlorinated biphenyl (Aroclor 1254) inhibits testosterone biosynthesis and antioxidant enzymes in cultured rat Leydig cells. Reproductive toxicology (Elmsford, N.Y.)..

[27] Dominiak A, Wilkaniec A, Wroczynski P, Jesko H, Adamczyk A (2016). Protective Effects of Selol Against Sodium Nitroprusside-Induced Cell Death and Oxidative Stress in PC12 Cells. Neurochemical research..

[28] Pinter E, Pozsgai G, Hajna Z, Helyes Z, Szolcsanyi J (2014). Neuropeptide receptors as potential drug targets in the treatment of inflammatory conditions. British journal of clinical pharmacology..

[29] Oba S, Hino M, Fujita T (2008). Adrenomedullin protects against oxidative stress-induced podocyte injury as an endogenous antioxidant. Nephrology, dialysis, transplantation: official publication of the European Dialysis and Transplant Association - European Renal Association..

[30] Ashizuka S, Inatsu H, Inagaki-Ohara K, Kita T, Kitamura K (2013). Adrenomedullin as a potential therapeutic agent for inflammatory bowel disease. Current protein & peptide science..

[31] Saeed Y (2014). Indirect effects of radiation induce apoptosis and neuroinflammation in neuronal SH-SY5Y cells. Neurochemical research..

[32] Hu W, Zhou PH, Zhang XB, Xu CG, Wang W (2015). Plasma concentrations of adrenomedullin and natriuretic peptides in patients with essential hypertension. Experimental and therapeutic medicine..

[33] Hung S-L, Lee N-G, Chang L-Y, Chen Y-T, Lai Y-L (2014). Stimulatory Effects of Glucose andPorphyromonas gingivalisLipopolysaccharide on the Secretion of Inflammatory Mediators From Human Macrophages. Journal of Periodontology..

[34] Xu K (2015). Autophagy attenuates the catabolic effect during inflammatory conditions in nucleus pulposus cells, as sustained by NF-kappaB and JNK inhibition. International journal of molecular medicine..

[35] Arulselvan P (2016). Role of Antioxidants and Natural Products in Inflammation. Oxidative medicine and cellular longevity..

[36] Amir Aslani B, Ghobadi S (2016). Studies on oxidants and antioxidants with a brief glance at their relevance to the immune system. Life sciences..

[37] Kopalli SR (2015). Korean red ginseng extract rejuvenates testicular ineffectiveness and sperm maturation process in aged rats by regulating redox proteins and oxidative defense mechanisms. Experimental gerontology..

[38] Eid AH, Abdelkader NF, Abd El-Raouf OM, Fawzy HM, El-Denshary ES (2016). Carvedilol alleviates testicular and spermatological damage induced by cisplatin in rats via modulation of oxidative stress and inflammation. Archives of pharmacal research..

[39] Shang T (2011). Toll-like receptor-initiated testicular innate immune responses in mouse Leydig cells. Endocrinology..

[40] Hedger M, Klug J, Frohlich S, Muller R, Meinhardt A (2005). Regulatory cytokine expression and interstitial fluid formation in the normal and inflamed rat testis are under leydig cell control. Journal of andrology..

[41] Le Goffic R (2002). Production of the chemokines monocyte chemotactic protein-1, regulated on activation normal T cell expressed and secreted protein, growth-related oncogene, and interferon-gamma-inducible protein-10 is induced by the Sendai virus in human and rat testicular cells. Endocrinology..

[42] Wu H (2016). Mumps virus-induced innate immune responses in mouse Sertoli and Leydig cells. Scientific reports..

[43] Abbas S (2016). UVB exposure enhanced benzanthrone-induced inflammatory responses in SKH-1 mouse skin by activating the expression of COX-2 and iNOS through MAP kinases/NF-kappaB/AP-1 signalling pathways. Food and chemical toxicology: an international journal published for the British Industrial Biological Research Association..

[44] Ma JQ, Ding J, Zhang L, Liu CM (2014). Ursolic acid protects mouse liver against CCl4-induced oxidative stress and inflammation by the MAPK/NF-kappaB pathway. Environmental toxicology and pharmacology..

[45] Mitchell S, Vargas J, Hoffmann A (2016). Signaling via the NFkappaB system. Wiley interdisciplinary reviews. Systems biology and medicine..

[46] Jeong HJ (2014). Down-regulation of MAPK/NF-kappaB signaling underlies anti-inflammatory response induced by transduced PEP-1-Prx2 proteins in LPS-induced Raw 264.7 and TPA-induced mouse ear edema model. International immunopharmacology..

[47] Hu W (2015). Adrenomedullin attenuates interleukin-1beta-induced inflammation and apoptosis in rat Leydig cells via inhibition of NF-kappaB signaling pathway. Experimental cell research..

