# Supplementary material for: Adrenomedullin protects Leydig cells against lipopolysaccharide-induced oxidative stress and inflammatory reaction via MAPK/NF-κB signalling pathways
Source: Sci Rep. 2017 Nov 28;7:16479. doi: 10.1038/s41598-017-16008-x (PMC5705677; doi:10.1038/s41598-017-16008-x)
Supplement: Supplementary file 1 — Dataset 7 and 8 [file 41598_2017_16008_MOESM1_ESM.doc]

# Adrenomedullin protects Leydig cells against lipopolysaccharide-induced oxidative stress and inflammatory reaction via MAPK/NF-κB signalling pathways

Wei Hu^1#^, Lei Shi^2#^, Ming-yong Li^3^*, Pang-hu Zhou^4^*, Bo Qiu^4^*, Ke Yin^5^, Hui-hui Zhang^3^, Yong Gao^6^, Ran Kang^3^, Song-lin Qin^1^, Jin-zhuo Ning^7^, Wei Wang^8^, Li-jun Zhang^9^

Author Affiliations

Departments of Andrology^1^, Urology^3^ and Orthopedics^5^, the First Affiliated Hospital of University of South China, No. 69 Chuan Shan Road, Hengyang 421001, Hunan Province, China

Departments of Oncology^2^, Orthopedics^4^ and Urology^7^, Renmin Hospital of Wuhan University, No. 238 Liberation Road, Wuhan, 430060, Hubei Province, China

Reproductive Medicine Centre^6^, the First Affiliated Hospital of Sun Yat-sen University, No. 58 Second Zhongshan Road, Guangzhou 510080, Guangdong Province, China

Department of Urology^8^, the First Affiliated Hospital of Anhui Medical University, No. 218 Jixi Road, Hefei 230022, Anhui Province, China

Department of Urology^9^, Minda Hospital Affiliated to Hubei Institute for Nationalities, No. 2 Wufengshan Road, Enshi 445000, Hubei Province, China

*Corresponding author: Ming-yong Li

Email address: limingyongdoctor@126.com

*Co-corresponding author: Pang-hu Zhou

Email address: zhoupanghu@126.com

*Co-corresponding author: Bo Qiu

Email address: qiuboprofessor@163.com

# Wei Hu and Lei Shi contributed equally to this work.


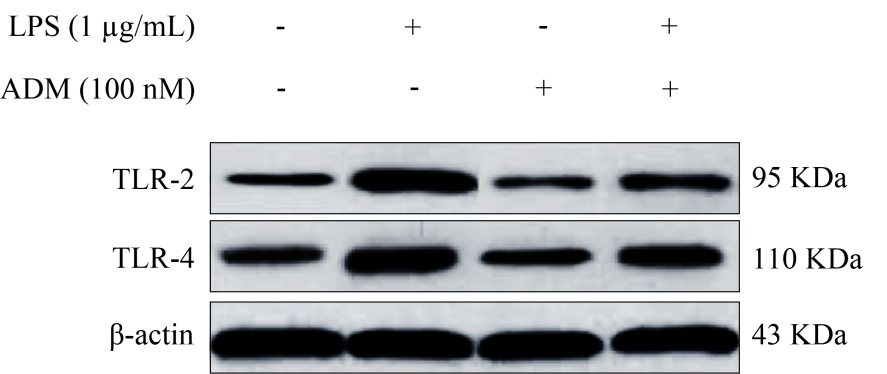


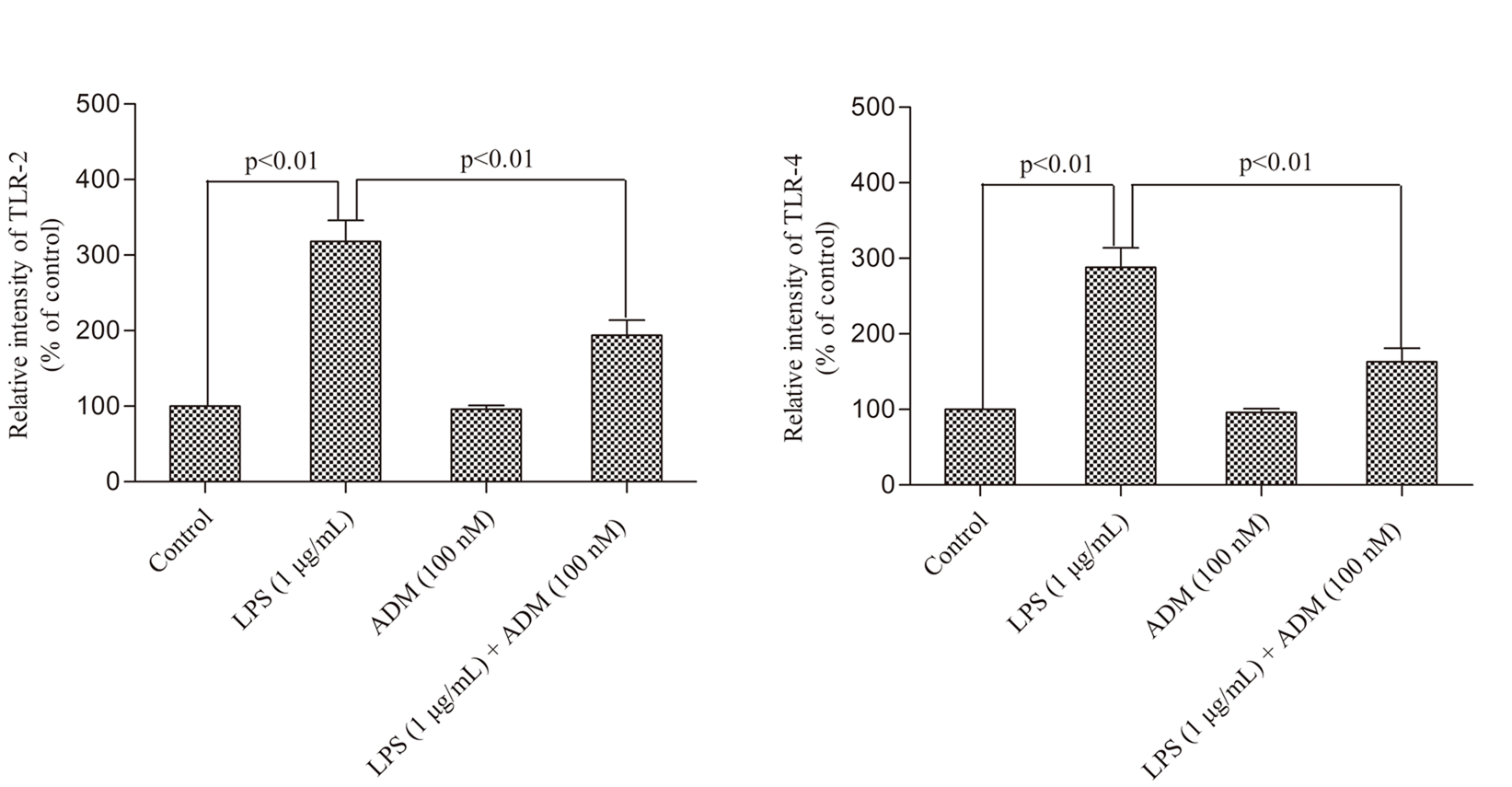


Effect of ADM on the LPS-induced production of TLR2 and TLR4 in primary Leydig cells in different treated groups and statistical analysis of Western blot results. β-actin was used as internal reference. Data were obtained from five independent experiments performed in triplicate and expressed as mean ± SEM.


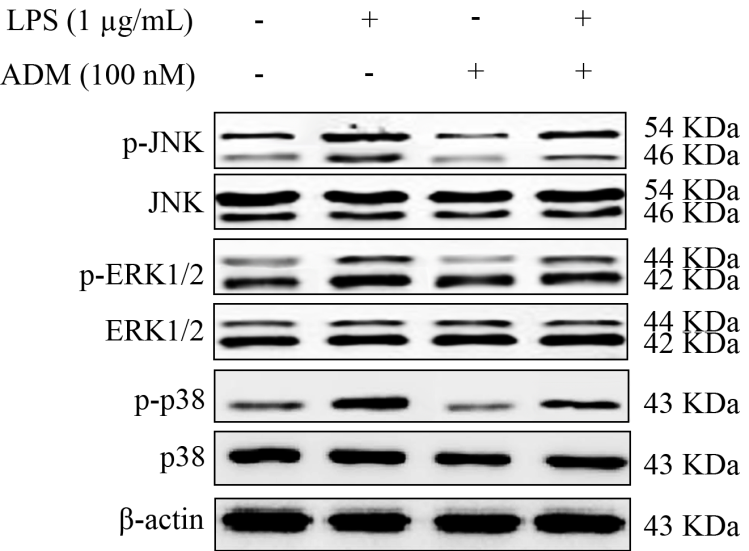


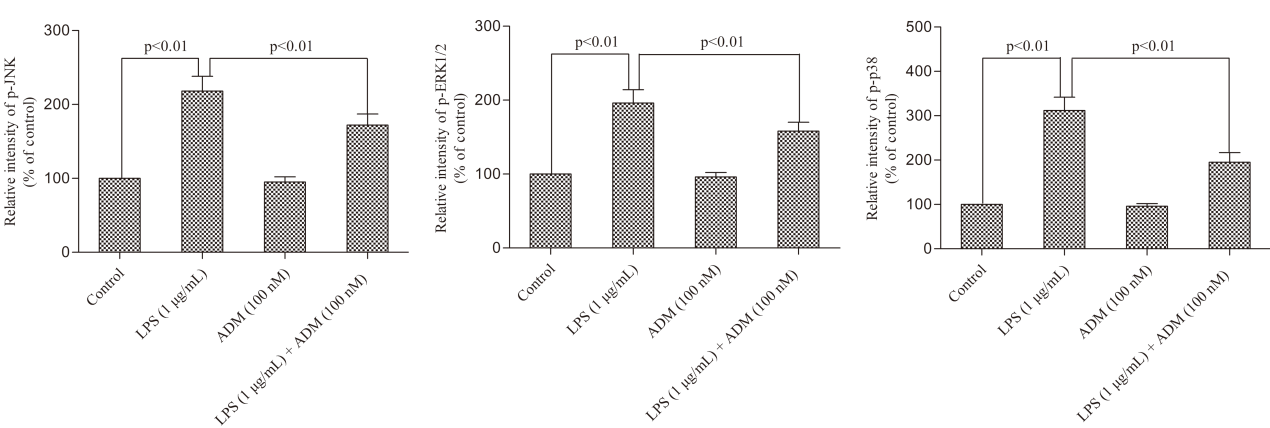


Effect of ADM on the LPS-induced phosphorylation of JNK, ERK1/2 and p38 in primary Leydig cells in different treated groups and statistical analysis of Western blot results. β-actin was used as internal reference. Data were obtained from five independent experiments performed in triplicate and expressed as mean ± SEM.


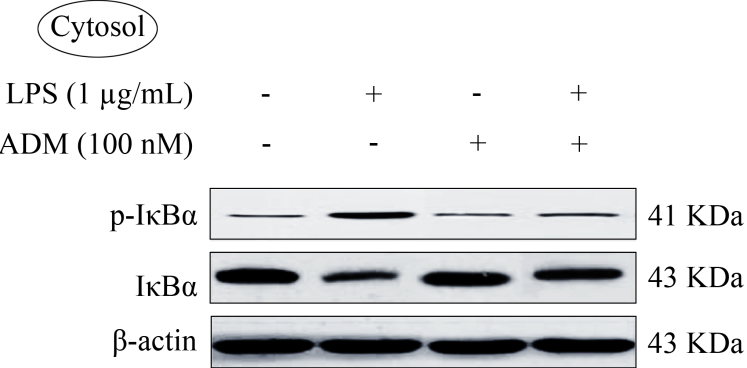


*
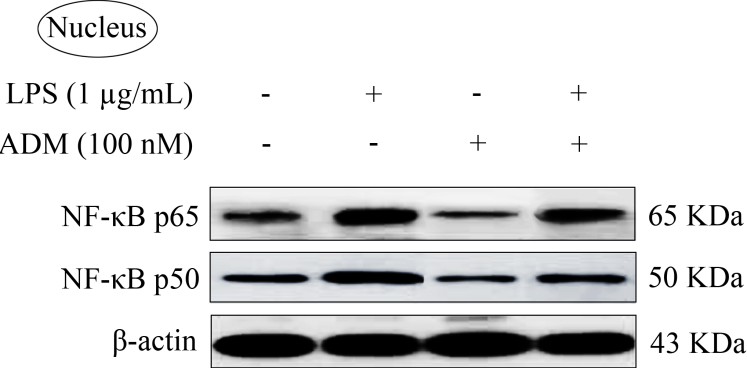
*


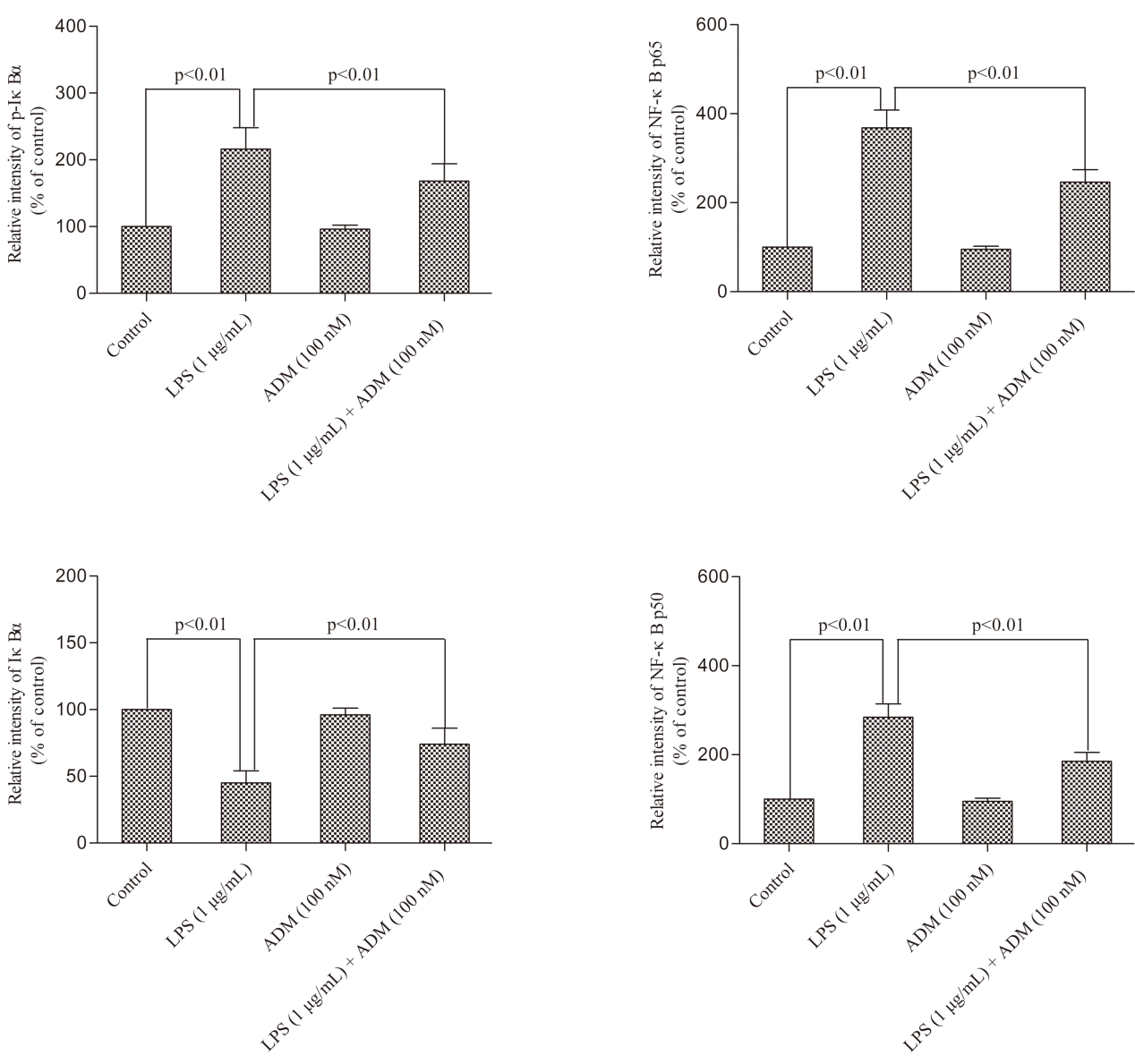


Effect of ADM on the LPS-induced p-IκBα in the cytoplasm and p-p65 and p-p50 in the nucleus in primary Leydig cells in different treated groups and statistical analysis of Western blot results. β-actin was used as internal reference. Data were obtained from five independent experiments performed in triplicate and expressed as mean ± SEM.
